# Integrating emergency medicine-directed urgent care into a federally qualified health center: case report from a health professional shortage area

**DOI:** 10.1186/s12913-026-15004-0

**Published:** 2026-06-26

**Authors:** Gavriel Brody, Luke Blumenberg, Joshua Gildin

**Affiliations:** ¹Aizer Health Center, Kiryas Joel, New York, USA

**Keywords:** Federally qualified health centers, Urgent care, Emergency medicine, Health professional shortage areas, Integrated care, Access to care, Community health centers

## Abstract

**Background:**

Underserved communities face significant barriers to healthcare access, often resulting in reliance on emergency departments (EDs) for non-emergent conditions. Federally Qualified Health Centers (FQHCs) play a key role in addressing these disparities, though many lack integrated urgent care services tailored to the needs of specific populations.

**Methods:**

This case study describes the implementation of an emergency medicine–directed urgent care department within an FQHC serving a predominantly Ultra-Orthodox Hasidic Jewish community in Kiryas Joel, New York. This report is intended as an implementation-focused description of how urgent care services can be integrated alongside primary care within an FQHC, rather than as a causal evaluation of ED use, clinical outcomes, or primary care utilization. A descriptive analysis of de-identified patient encounter data from 2020 to 2025 was conducted to characterize patient volume and utilization patterns.

**Results:**

From 2020 to 2025, patient volume increased from 1,215 to 13,636 encounters, representing a 1022.3% increase. Pediatric patients accounted for 65.1% of visits, comprising the majority of encounters. The department provides extended hours, holiday accessibility, and integrated referral pathways, supporting high utilization in a community with significant cultural and linguistic barriers.

**Conclusions:**

This case study suggests that an emergency medicine–directed urgent care model can be successfully implemented within an FQHC and achieve high utilization in an underserved population. While causal impacts on emergency department utilization, primary care utilization, continuity of care, and clinical outcomes were not evaluated, this model offers a practical framework for health centers seeking to expand same-day urgent care access within an integrated FQHC setting.

## Introduction

In underserved communities, healthcare access is frequently constrained by economic, social, and systemic barriers. Challenges such as financial limitations, shortages of primary care providers, language barriers, and inadequate transportation disproportionately affect these populations. Consequently, millions of individuals living in primary care Health Professional Shortage Areas (HPSAs) lack sufficient primary care access, relying instead on emergency departments (EDs) for medical needs, even when conditions are non-urgent [1, 2]. This overreliance on EDs not only exacerbates overcrowding but also increases healthcare costs for an already socioeconomically disadvantaged population [3]. When patients seek care in the ED rather than with primary care physicians who have a comprehensive understanding of their medical history, they may miss opportunities for continuity of care [4]. One study estimated that 27% of ED visits could be managed in urgent or primary care settings, with potential savings of $4.4 billion annually [5]. 

Federally Qualified Health Centers (FQHCs) are pivotal in addressing healthcare disparities by accepting Medicaid and Medicare and delivering comprehensive primary care services to underserved populations [6, 7]. However, prior literature has identified persistent gaps in same-day access and urgent care capacity within FQHCs, which may limit their ability to manage time-sensitive, non-emergent conditions and contribute to continued reliance on ED services. Existing studies have explored patient decision-making in seeking urgent and emergency care, as well as system-level strategies to reduce ED utilization, including enhanced primary care access and care coordination [2, 5]. Despite these efforts, there remains limited published literature describing the integration of urgent care services directly within FQHC settings—particularly models staffed by emergency medicine physicians and adapted to meet the cultural and linguistic needs of specific underserved populations.

In addition, while culturally competent care has been recognized as an important factor influencing healthcare access and utilization, few studies have described how urgent care delivery models can be tailored to align with the sociocultural and religious needs of distinct communities. Standard healthcare delivery models may not adequately address barriers related to language, health literacy, or cultural practices, which can influence when and where patients seek care.

This case study describes the implementation of an emergency medicine–directed urgent care department within an FQHC serving a predominantly Ultra-Orthodox Hasidic Jewish community in Kiryas Joel, New York. Rather than evaluating causal changes in emergency department utilization, clinical outcomes, continuity of care, or primary care utilization, this study provides an implementation-focused description of the structure, services, and utilization patterns of this integrated care model. Specifically, this case study sought to describe how the urgent care model was structured within the FQHC, characterize early utilization patterns, and identify operational features that may inform similar implementation efforts in other underserved settings. By characterizing how urgent care services were embedded alongside primary care within a real-world FQHC setting, with particular attention to culturally tailored features, this work contributes to the limited body of literature on urgent care integration within FQHCs and offers a practical framework that may inform similar efforts in other underserved settings.

## Methods

### Study design

This study employed a descriptive case study methodology to characterize the implementation and utilization of an emergency medicine–directed urgent care department within a Federally Qualified Health Center (FQHC). Case study methodology is commonly used in healthcare research to provide in-depth examination of complex service delivery models within real-world contexts, particularly when evaluating systems-level interventions where experimental designs are not feasible [8]. This approach was selected to describe the operational structure, services, and patient utilization patterns of the Aizer Urgent Care Department, an emergency medicine–directed urgent care service embedded within Aizer Health Center, an FQHC in Kiryas Joel, New York, not to evaluate causal effects on healthcare outcomes. Therefore, utilization trends were interpreted as indicators of implementation feasibility, service demand, and community uptake, rather than as evidence of causal effects on emergency department use or clinical outcomes.

### Setting and population

The study was conducted at Aizer Health Center in Kiryas Joel, New York, a predominantly Ultra-Orthodox Hasidic Jewish community in Orange County. Kiryas Joel has experienced rapid population growth, reaching approximately 43,863 residents in 2024 from 32,960 in 2020, and has one of the youngest median ages in the United States (14.8 years), reflecting high birth rates and large family sizes [9, 10]. 

The community faces significant socioeconomic and linguistic barriers to healthcare access. Approximately 40% of residents live below the federal poverty line, and the majority of the population (92.65%) speaks Yiddish as a primary language [9, 11]. These factors, combined with high population density, contribute to challenges in obtaining timely medical care [12]. 

Kiryas Joel is designated as both a Health Professional Shortage Area (HPSA) and a Medically Underserved Area (MUA). HPSA designation, defined by the Health Resources and Services Administration (HRSA), indicates a shortage of primary care providers relative to the population, while MUA designation reflects a broader index incorporating provider availability, poverty levels, elderly population, and infant health outcomes [13]. These overlapping designations highlight both workforce shortages and broader structural barriers to care access within the community.

Publicly available demographic and socioeconomic data, including U.S. Census Bureau, Data USA, World Population Review, and HRSA shortage-designation data, were reviewed to contextualize community-level barriers. These public data were used only to describe the broader community context; utilization measures reported in Tables 1 and 2 were obtained from Aizer Urgent Care Department internal encounter data.

### Intervention: urgent care model

The Aizer Urgent Care Department was established in July 2020 to address gaps in timely access to care within the community. Initially, the department operated on a limited after-hours schedule from 8 PM to 12 AM to provide coverage when primary care services were unavailable. Over time, in response to increasing patient demand and institutional needs within the FQHC, operating hours were progressively expanded to the current schedule of 9 AM to 12 AM Monday–Thursday and evenings on Sundays. This increase in patient volume occurred alongside expanded service availability. Currently, the department is staffed by 14 emergency medicine physicians, including pediatric emergency specialists.

The department was developed as a complementary service line within the FQHC rather than as a replacement for primary care. Primary care services continued to provide preventive care, chronic disease management, routine follow-up, and longitudinal care, while the Urgent Care Department focused on same-day access, acute complaints, minor procedures, diagnostic evaluation, and time-sensitive clinical needs. This structure allowed urgent care services to be embedded within the FQHC while remaining connected to the broader primary care infrastructure.

The department provides a broad range of diagnostic and procedural services, including point-of-care laboratory testing, laceration repair, incision and drainage, foreign body removal, and diagnostic imaging for acute conditions. In addition, the model incorporates operational features designed to improve access within the local context, including walk-in availability, proximity within walking distance for many residents, and integration with in-house specialty and primary care services.

To support care coordination, the department utilizes a structured referral system and follow-up protocol. Follow-up telephone encounters were defined as documented post-visit phone contacts by the care team for selected patients, such as post-procedural or clinically complex cases, to facilitate reassessment, reinforce care plans, and coordinate additional follow-up when needed. In this study, the proportion of patients receiving follow-up telephone encounters within three days was measured as a descriptive indicator of care processes.

### Integrated care features

In addition to core urgent care services, the Urgent Care Department incorporates several integrated care features designed to align with the needs of the population served. These include workflows such as allowing for scheduling of overdue pediatric preventive visits during urgent care encounters when families are already present in the department.

Communication with adult and pediatric primary care departments is facilitated through HIPAA-compliant messaging systems to support coordination of follow-up care for patients requiring ongoing management. Patients requiring specialty evaluation, including podiatry, dermatology, cardiology, pediatric otolaryngology, optometry, and pulmonology, may be referred directly to in-house specialty services through established referral pathways.

The department also provides access to on-site behavioral health clinicians, allowing for same-day evaluation and support when clinically indicated. Behavioral health clinicians are typically available on-site until 8:00 PM and can be engaged through a structured “warm handoff” protocol, in which urgent care providers electronically notify behavioral health staff to facilitate prompt, real-time clinical evaluation. In addition, bidirectional referral patterns exist between primary care and urgent care services, with primary care providers referring patients to the Urgent Care Department for procedural or time-sensitive evaluations.

Most recently, the department implemented point-of-care high-sensitivity troponin testing for selected low-risk chest pain presentations as part of its urgent care diagnostic capabilities.

The model was also designed around local cultural, linguistic, and logistical needs. The department is located in the center of town, making urgent care physically accessible for many residents. Interpreter services were used when needed to support communication with patients and families in a community where most residents speak Yiddish as a primary language. In addition, holiday hours were offered during periods when other local health facilities were closed for religious observance, helping maintain access to urgent care during times when routine healthcare availability may otherwise be limited.

### Data sources and measures

This study analyzed aggregated, de-identified patient encounter data obtained from the Aizer Urgent Care Department’s internal data systems. The dataset included the following variables:

- Total annual patient volume (2020–2025).
- Patient demographics (age and gender).
- Visit type (scheduled vs. walk-in).
- Volume of visits during major Jewish holidays.
- Proportion of encounters with follow-up telephone contact within three days.

These measures were selected to characterize utilization patterns, access to care, and operational workflows within the urgent care model. Publicly available demographic data were used to contextualize observed utilization patterns within the broader socioeconomic and cultural environment of the community.

### Data analysis

Descriptive statistical analyses were performed to summarize patient utilization and operational trends. Annual patient volume was reported as total encounter counts per year, and percent change in volume was calculated using the formula:

$$\begin{aligned}&\:Percent\:Change\cr &=\frac{Final\:Year\:-\:Initial\:Year}{Initial\:Year}\:\times\:\:100\end{aligned}$$

Patient demographics and visit characteristics were reported as proportions. For holiday-related analyses, patient volume was examined across calendar years, with consideration of the number of days associated with each holiday period to allow interpretation of utilization trends. Descriptive analyses were performed using Microsoft Excel.

All analyses were conducted using aggregated data, and no inferential statistical testing was performed, consistent with the descriptive nature of the case study.

### Ethical considerations

This study was reviewed by WCG IRB (WCG Clinical) and was granted exempt status under 45 CFR § 46.104(d)(4). The requirement for informed consent was waived due to the use of retrospective, aggregated, and de-identified data, with no participant contact or re-identification. All procedures were conducted in accordance with the ethical standards of the Declaration of Helsinki.

## Results

### Patient volume

A total of 52,004 encounters were recorded in the Urgent Care Department between 2020 and 2025. Annual encounter volume increased from 1,215 in 2020 to 13,636 in 2025, representing a 1022.3% increase over the study period. Growth was most pronounced during the early implementation period, rising from 1,215 encounters in 2020 to 8,979 in 2022, followed by continued increases through 2025. Annual encounter volume and visit-characteristic data are summarized in Table 1. Because this growth occurred alongside expanded operating hours and local population growth, these data are interpreted descriptively as evidence of increasing utilization, community uptake, and service demand rather than as a causal measure of reduced emergency department use or improved clinical outcomes.

### Patient demographics

Pediatric patients accounted for the majority of encounters (65.1%), followed by adults aged 18–65 (32.8%) and geriatric patients (2.0%). The gender distribution of encounters was relatively balanced, with 51.1% involving male patients and 48.9% involving female patients. These demographic characteristics are summarized in Table 1.

### Visit characteristics

Scheduled visits comprised 59.7% of encounters, while 40.3% were classified as walk-in visits. Follow-up telephone encounters documented within 3 days occurred in 19,241 of 52,004 encounters (37.0%). These visit characteristics are summarized in Table 1.

### Holiday utilization

Patient utilization during covered Jewish holidays increased over time, indicating sustained demand for urgent care services during periods when routine healthcare access may be more limited. Aggregate holiday-related visit volume increased from 256 encounters in 2020 to 2,077 encounters in 2025 (approximately a 711% increase). Detailed holiday-specific volumes are presented in Table 2. Because the department launched on July 13, 2020, holiday data for 2020 reflect a partial year and exclude several holidays that occurred before implementation. Additionally, the department was closed on Tzom Gedaliah in 2024 and Purim in 2024 and 2025, which is reflected in the corresponding volume data.

**Table 1:** Summary of encounter volume, patient demographics, and visit characteristics, 2020–2025

Category	Variable	*n* (%) or total encounters
Encounter volume	Total encounters (2020–2025)	52,004
	2020	1,215
	2021	5,723
	2022	8,979
	2023	11,091
	2024	11,360
	2025	13,636
Age distribution	Pediatric, 0–18 years	65.10%
	Adult, 18–65 years	32.80%
	Geriatric, > 65 years	2.00%
Gender distribution	Male	51.10%
	Female	48.90%
Visit type	Scheduled	59.70%
	Walk-in	40.30%
Follow-up telephone encounters	Within 3 days	19,241 / 52,004 (37.0%)

**Table 2:** Patient utilization during covered Jewish holidays, 2020–2025

		Year					
Holiday	Number of Days	2020	2021	2022	2023	2024	2025
Rosh Hashanah	2	20	72	120	121	141	140
Tzom Gedaliah	1	17	44	42	51	0	75
Yom Kippur	1	16	39	31	51	43	41
Sukkot	7	78	198	314	395	306	454
Hanukkah	8	118	394	378	257	271	522
Taanit Esther	1	0	11	16	21	59	35
Purim	1	0	11	23	37	0	0
Passover	8	0	121	147	217	364	592
Shavuot	2	0	27	87	134	125	132
Tisha B'Av	1	7	0	0	98	81	86
Total Encounters		256	917	1158	1382	1390	2077

## Discussion

This case study describes the implementation and early utilization patterns of an emergency medicine–directed urgent care department embedded within a Federally Qualified Health Center (FQHC) serving a predominantly Ultra-Orthodox Hasidic Jewish community in Kiryas Joel, New York. The primary contribution of this report is its description of how urgent care services can be integrated alongside primary care within an FQHC, rather than its ability to measure causal outcomes. Several findings are notable. First, encounter volume increased substantially over the study period, from 1,215 visits in 2020 to 13,636 in 2025, suggesting strong community uptake of the service after implementation. Second, pediatric patients accounted for the majority of encounters, consistent with the community’s young age structure and large family size [9, 10]. Third, the department maintained use across both scheduled and walk-in visits, indicating demand for both planned and same-day access to urgent care. Finally, utilization during covered Jewish holidays increased over time, suggesting that the department was used during periods when routine access to care may otherwise be more limited [14, 15]. 

These findings align with prior literature showing that underserved populations often face barriers to timely access to primary and urgent care, including provider shortages, transportation challenges, and language-related barriers, which may contribute to reliance on emergency departments for non-emergent conditions [1–5, 12]. FQHCs play an important role in addressing these disparities, yet published literature describing urgent care integration within FQHC settings remains limited [6, 7]. In that context, this study contributes a descriptive example of how an FQHC-based urgent care model can be structured to meet local access needs while remaining connected to primary care, specialty, and behavioral health services.

The predominance of pediatric encounters is particularly important in interpreting the role of this department. Kiryas Joel has an unusually young population, and the observed age distribution of visits suggests that acute pediatric needs represent a major component of healthcare demand in this setting [9, 10]. This finding may have operational implications for other community-based urgent care models serving similarly young populations, including staffing decisions, pediatric emergency expertise, and the availability of same-day family-centered care.

The observed use of the department during Jewish holidays is also notable. In this community, religious observances and related restrictions may complicate access to routine healthcare services during certain periods [14, 15]. Increasing holiday-related utilization over time suggests that the department functioned as an accessible point of care during these intervals. Although this study cannot determine whether holiday availability reduces emergency department use, the pattern supports the practical importance of aligning service availability with community-specific needs.

More broadly, this case study highlights the potential value of tailoring urgent care delivery models to the sociocultural and logistical realities of the communities they serve. In this setting, the urgent care department was designed to operate within walking distance for many residents, accommodate a largely Yiddish-speaking population, and function within a community marked by both workforce shortages and broader indicators of medical underservice [9, 11–13]. While the present study does not evaluate patient satisfaction, continuity of care, or clinical outcomes, the high and increasing utilization observed over time suggests that the model addressed a meaningful access need.

### Limitations

The findings should be interpreted in light of several limitations. First, this was a single-site descriptive case study, which limits generalizability to other FQHCs and community settings [8]. Second, the analysis relied on aggregated operational data and did not include pre-implementation comparison data, emergency department utilization data, primary care utilization data, patient-reported outcomes, cost data, or longitudinal clinical outcomes. As a result, the study cannot determine whether the department reduced emergency department reliance, improved continuity of care, improved health outcomes, or affected primary care utilization within the FQHC. The study also cannot determine whether increased urgent care utilization represented new access, substitution for emergency department care, substitution for primary care visits, or a combination of these factors. Third, a period of electronic medical record (EMR) downtime led to incomplete capture of some patient encounters. In addition, follow-up telephone encounters were not always assigned the correct designation in the EMR, likely leading to underestimation of the actual follow-up rate. Some walk-in patients were also labeled administratively as scheduled visits, which may have affected the observed distribution of visit types. The available dataset also did not permit reliable breakdown of utilization by specific service type, such as procedures, imaging, point-of-care testing, behavioral health evaluation, or specialty referral. These documentation issues may have introduced measurement error, although they are unlikely to alter the overall pattern of increasing utilization and high service demand.

Despite these limitations, this case study offers a practical example of urgent care integration within an FQHC serving an underserved and culturally distinct population. The model may be relevant to other health centers seeking to expand access to urgent care while maintaining connection to broader primary and specialty care infrastructure [6, 7]. Future research should evaluate whether similar models affect emergency department utilization, primary care utilization, continuity of care, timeliness of follow-up, patient experience, cost, and clinical outcomes.

## Conclusions

This case study describes the implementation of an emergency medicine–directed urgent care department within a Federally Qualified Health Center serving a culturally distinct and medically underserved community. Over the study period, the department demonstrated substantial growth in utilization, with pediatric patients comprising the majority of encounters and continued use during holiday periods when routine access to care may be more limited. These findings suggest that an integrated urgent care model tailored to local community needs can serve as an important access point for urgent care within an FQHC setting.

Although this study does not evaluate causal effects on emergency department utilization, primary care utilization, continuity of care, clinical outcomes, or patient experience, it provides a practical example of how urgent care services can be embedded within a broader FQHC-based safety-net infrastructure. This model may inform health centers seeking to expand same-day urgent care access while maintaining connection to primary care, specialty care, behavioral health, and follow-up workflows.

## Data Availability

The datasets used and analyzed during the current study are available from the corresponding author on reasonable request.

## Abbreviations

ED: Emergency department
EMR: Electronic medical record
EMS: Emergency Medical Services
ENT: Ear, Nose and Throat
FQHC: Federally Qualified Health Center
HIPAA: Health Insurance Portability and Accountability Act
HPSA: Health Professional Shortage Area
HRSA: Health Resources and Services Administration
IRB: Institutional Review Board
MUA: Medically Underserved Area

## References

[1] Kona M, Houston M, Gooding N. The effectiveness of policies to improve primary care access for underserved populations: an assessment of the literature. 2022. https://www.milbank.org/wp-content/uploads/2022/01/Georgetown_6.pdf#page42.

[2] Coster JE, Turner JK, Bradbury D, Cantrell A. Why do people choose emergency and urgent care services? A rapid review utilizing a systematic literature search and narrative synthesis. Theodoro DL, ed. Acad Emerg Med. 2017;24(9):1137–1149. 10.1111/acem.13220. 10.1111/acem.13220 PMC5599959 28493626

[3] Uscher-Pines L, Pines J, Kellermann A, Gillen E, Mehrotra A. Deciding to visit the emergency department for non-urgent conditions: a systematic review of the literature. Am J Manag Care. 2013;19(1):47. https://pmc.ncbi.nlm.nih.gov/articles/PMC4156292/. 23379744 PMC4156292

[4] Rocovich C. Emergency department visits: Why adults choose the emergency room over a primary care physician visit during regular office hours? World J Emerg Med. 2012;3(2):91. 10.5847/wjem.j.issn.1920-8642.2012.02.002. 25215045 10.5847/wjem.j.issn.1920-8642.2012.02.002 PMC4129795

[5] Timmins L, Peikes D, McCall N. Pathways to reduced emergency department and urgent care center use: lessons from the comprehensive primary care initiative. Health Serv Res. 2020;55(6):1003–12. 10.1111/1475-6773.13579. 33258126 10.1111/1475-6773.13579 PMC7704466

[6] Healthcare.gov. Federally Qualified Health Center (FQHC) - HealthCare.gov Glossary. HealthCare.gov. Published 2024. https://www.healthcare.gov/glossary/federally-qualified-health-center-fqhc/.

[7] Rural Health Information Hub. Federally Qualified Health Centers (FQHCs) Introduction - Rural Health Information Hub. Ruralhealthinfo.org. Published 2025. https://www.ruralhealthinfo.org/topics/federally-qualified-health-centers.

[8] Baker GR. The contribution of case study research to knowledge of how to improve quality of care. BMJ Qual Saf [Internet]. 2011;20(Suppl 1):i30–5. Available from: https://qualitysafety.bmj.com/content/20/Suppl_1/i30. 10.1136/bmjqs.2010.046490 PMC3066793 21450767

[9] U.S. Census Bureau QuickFacts: Kiryas Joel village, New York. https://www.census.gov; https://www.census.gov. Accessed 25 Mar 2025.

[10] Data USA. Kiryas Joel, NY. Data USA; 2023. Available from: https://datausa.io/profile/geo/kiryas-joel-ny/. Accessed 25 Mar 2025.

[11] World Population Review. Kiryas Joel, NY population 2025. World Population Review; 2025. Available from: https://worldpopulationreview.com/us-cities/new-york/kiryas-joel. Accessed 25 Mar 2025.

[12] National Academies of Sciences. Factors That Affect Health-Care Utilization. National Academies Press (US); 2019. https://www.ncbi.nlm.nih.gov/books/NBK500097/.

[13] HRSA. What is Shortage Designation? | Bureau of Health Workforce. bhw.hrsa.gov. Published June 2023. https://bhw.hrsa.gov/workforce-shortage-areas/shortage-designation#mups.

[14] Halachic guidelines for accessing medical care on SHABBAT; 2024. https://chicagomitzvahcampaign.org/wp-content/uploads/2024/07/Accessing-medical-care-on-Shabbat-excerpted-from-the-Yeshuos-Directory-5784.pdf. Accessed 25 Mar 2025.

[15] Scheinman M. Sabbath observance and delayed primary repair of lacerations: experience from a plastic surgery practice. Plast Reconstr Surg Global Open. 2024;12(9):e6148–6148. 10.1097/gox.0000000000006148. 10.1097/GOX.0000000000006148 PMC11387041 39258278

